# Editorial: Verification, Falsification, and the Methodology Paradigm Shift for Scientific Psychology

**DOI:** 10.1002/pchj.70111

**Published:** 2026-06-22

**Authors:** Jianxin Zhang

**Affiliations:** ^1^ Institute of Psychology Chinese Academy of Sciences Beijing China

One might ask: Is the role of psychology in today's rapidly changing human life becoming increasingly insignificant? In recent years, artificial intelligence (AI) has developed rapidly and has had a tangible and significant impact on human life, yet the human intelligence studied by psychology seems to have lost its role “as a template for AI” that it held in the late twentieth century; the resolution of human mental disorders increasingly relies on discoveries in neuroscience, biochemistry, and genetics; psychology's solutions to everyday human psychological distress have become more diverse and fragmented, without demonstrating significant improvements in counseling and therapeutic efficacy; in the face of frequent disputes and even wars between nations, ethnic groups, and social classes around the world, psychology appears even more at a loss. The same is true of Chinese psychology: while the number of research papers published across various categories has skyrocketed, its overall performance in addressing the urgent problems facing contemporary China and humanity can be characterized as “quantity far exceeding quality,” with few disruptive new theories or widely applicable new methods emerging.

The new editorial board of *PsyCh Journal* is about to take office, and its members have been entrusted with great expectations and responsibilities. Everyone must strive to lead by example, and encourage and guide authors to broaden their horizons, dare to innovate, and make the discovery of new problems, exploration of new fields, construction of new theories, and provision of new tools the primary goals of their careers; they must genuinely redirect their research perspectives toward addressing and solving real‐world human problems. Psychology researchers should no longer be content with continuously producing articles that merely scratch the surface.

There are many reasons for the lack of innovation in psychological research. However, researchers' insufficient sensitivity to changes in scientific methodology and its paradigms may be one of the important causes. For over a century, science has been evolving and is by no means static. Methodology has gradually advanced from initial logical positivism to Popper's falsificationism and has subsequently been encompassed by the Duhem–Quine thesis, thereby elevating reductionist falsification to holistic falsification. The evolution of methodology has enhanced scientists' awareness of theoretical innovation, thereby promoting the rapid development of science as a whole, but psychology seems to have lagged behind in this regard.

As the new Editor‐in‐Chief of *PsyCh Journal*, this editorial revisits the philosophical origins, core distinctions, complementary roles, and practical implications of the methodologies of verification and falsification, and outlines a vision for advancing critical, rigorous, and innovative scientific research of psychology.

Scientific psychology, as an integral part of modern science, must abide by evolving scientific methodologies. As Thomas Kuhn argued, science advances through periodic paradigm shifts, and the transition from logical positivism to Popperian falsificationism represents one of the most significant leaps in modern scientific thought. For decades, psychological research has been deeply rooted in a verification‐dominated culture. Researchers are systematically trained to propose directional hypotheses, design studies to support those hypotheses, and rely on significance testing, model fitting, and explanatory power to confirm theoretical expectations. Studies yielding significant, supportive results are readily published, while those producing null or negative findings are often labeled “failed” and confined to the “file drawer.” Over time, this culture generates publication bias, reinforces confirmation bias, discourages critical self‐reflection, and impedes genuine theoretical evolution.

Verification, rooted in 20th‐century logical positivism, holds that scientific knowledge derives exclusively from sensory experience and inductive reasoning. Its core principle is that meaningful scientific statements must be verifiable through empirical observation; propositions that cannot be verified empirically are meaningless non‐scientific statements. In practice, verification seeks to accumulate positive evidence, refine existing theories, and expand their explanatory scope. This approach has helped establish foundational psychological phenomena and construct initial theoretical frameworks. However, verification carries an irreparable logical flaw: no finite number of positive observations can definitively prove a universal proposition. Inductive reasoning can strengthen confidence in a theory, but it can never establish it as absolute truth. Overreliance on verification fosters theoretical conservatism and leaves core assumptions unchallenged.

Against these limitations, Karl Popper's falsificationism provides a revolutionary foundation for scientific methodology. Falsification rests on deductive logic: universal scientific theories cannot be verified, but they can be decisively rejected by a single reliable counterexample—the well‐known “black swan effect.” Falsifiability thus becomes the defining criterion that separates science from pseudoscience. Genuine scientific theories must explicitly state conditions under which they can be refuted and tested; theories that endlessly adapt to avoid refutation are not scientific. In practice, falsification shifts the research mission from “proving a theory correct” to “testing whether a theory can be wrong.” Under this paradigm, theories are not fixed truths but provisional, testable conjectures that remain valid only until replaced by better explanations. This mindset encourages researchers to design rigorous, even “destructive” tests, explore boundary conditions, and engage seriously with null and negative results.

Verification‐oriented studies aim to maximize support for theoretical hypotheses, by controlling for extraneous variables, and prioritizing confirmatory evidence. While falsification‐oriented studies actively seek conditions that may challenge or limit theoretical claims, they simultaneously emphasize statistical power.

For example, using structural equation modeling results that fulfill general fit criteria indicating mindfulness reduces anxiety. This typical example reflects that verification methods are not used to judge the truth or falsity of theories, but to evaluate the rationality and adaptability of models, thereby providing empirical support for theories through data fitting. On the other hand, the result is interpreted cautiously under falsification: researchers acknowledge untested boundary conditions and design future studies to identify limitations. Falsification thinking focuses on the testing of null hypotheses, emphasizing the analysis of experimental errors, statistical power, and result reliability. A non‐significant result is not dismissed; instead, researchers evaluate sample size, effect size, and statistical power to distinguish between a true null effect and a failed detection. Bayesian statistical indicators are checked to quantify support for different significance of the null hypothesis. Bayesian analysian has been more frequently today to further clarify whether data represent “no evidence for an effect” or “evidence for no effect,” restoring scientific value of null hypotheses once discarded.

In summary, verification and falsification are essentially different in five dimensions: research logic, experimental design, data interpretation, theoretical attitude, and application scenarios.

- Research Logic Difference: Inductive Accumulation vs. Deductive Refutation

Verification thinking takes inductive logic as its core, following the research path of “specific observation–summary of laws–theory improvement,” suitable for research scenarios of initial theory construction and phenomenon exploration. Falsification thinking takes deductive logic as its core, following the research path of “hypothesis formulation–deductive prediction–test and refutation.” This logic breaks through the limitations of induction, capable of negating universal propositions with a single counterexample, possessing stronger theoretical innovation capabilities.

- Research Design Difference: Positive Support vs. Reverse Testing

Verification research design is oriented to “collecting positive evidence,” focusing on optimizing experimental procedures, controlling irrelevant variables, and maximizing the fit between data and hypotheses. Common methods include questionnaire surveys, regression analysis, exploratory case studies, and historical data induction, with the core goal of supplementing empirical support for existing theories. Falsification research design is oriented to “searching for refuting evidence,” actively avoiding research bias, designing rigorous testing schemes, and even conducting “destructive experiments” to challenge existing theories. The main methods of falsification research include randomized controlled trials, double‐blind experiments, competitive hypothesis testing, and predictive validation modeling, with the core goal of finding key factors, conditions, and boundaries that can challenge or even overturn existing theories.

- Data Interpretation Difference: Focus on Fitting vs. Focus on Reflection

Verification thinking focuses on positive indicators such as regression coefficients, model fit, and explained variance, with the core question: “Does the data support the research hypothesis?” This interpretation mode easily leads to confirmation bias, where researchers often ignore abnormal data and non‐significant results, selectively accepting positive evidence, showing insensitivity in data analysis and conservatism in theory construction. Falsification thinking focuses on unexpected results with the core logic: “Can the data overturn the existing hypothesis (and its underlying theory)?” Researchers actively report invalid and negative results, deeply analyze the causes of non‐significant results, distinguish between theoretical flaws and experimental design defects, minimizing research bias on the one hand, and showing the courage to break the constraints of existing theories to innovate new models and theories that include counterexamples on the other.

- Theoretical Attitude Difference: Conservative Improvement vs. Critical Innovation

Verification thinking holds a conservative and inclusive attitude toward existing theories, taking established theories as the core framework, and continuously supplementing boundary conditions and refining details to adapt theories to more research scenarios, essentially protecting and expanding existing theories. Falsification thinking holds an open and critical attitude toward theories, rejecting absolute truth and regarding all scientific theories as “conjectures not yet overturned.” Once empirical data continuously refute theoretical hypotheses, researchers take the initiative to abandon old theories and construct new ones. Therefore, falsification thinking becomes a higher‐level methodology for disciplinary theoretical iteration, breakthrough innovation, and scientific progress. The Duhem–Quine thesis reminds us that hypotheses are never tested in isolation: unexpected results may reflect flaws in auxiliary assumptions, measurement tools, or research design rather than the core theory itself. Falsification therefore functions as holistic, iterative criticism that strengthens the entire theoretical network rather than simply rejecting individual claims.

A mature science requires both: the constructive accumulation of verification and the critical rigor of falsification. Innovation arises not from repeating supportive results, but from courageously challenging existing assumptions, exploring negative evidence, and revising theories to accommodate new observations.

Our vision is to make *PsyCh Journal* a leading platform for transformative psychological science that is cumulative yet self‐correcting, empirical yet critical, and globally influential yet deeply rooted in methodological integrity. Verification constructs; falsification renews. By embracing both, we will build a more robust, innovative, and credible science of psychology.

Psychology needs a flagship publication that rewards not only critical thinking, methodological pluralism, and theoretical boldness, but also human being's interest and benefits‐oriented. We will foster a truly inclusive, global intellectual community that bridges diverse research traditions, encourages cross‐cultural dialogue, and elevates collaboration that makes psychology become problem‐sensitive, technology‐leading, life‐improving, and theoretically innovative.

## Conflicts of Interest

The author declares no conflicts of interest.

## Data Availability

Data sharing not applicable to this article as no datasets were generated or analysed during the current study.

